# A Rare Case of Pseudo-atrial Flutter Waves in a Patient with Essential Tremor

**DOI:** 10.7759/cureus.3934

**Published:** 2019-01-21

**Authors:** Christos A Papanastasiou, Dimitrios Petroglou, Leonidas Palaiodimos, Fotios Economou

**Affiliations:** 1 Cardiology, Aristotle University - School of Medicine, Thessaloniki, GRC; 2 Cardiology, 424 General Military Hospital, Thessaloniki, GRC; 3 Internal Medicine, Montefiore Medical Center / Albert Einstein College of Medicine, New York, USA

**Keywords:** atrial flutter, essential tremor

## Abstract

High-frequency muscle tremor can mimic atrial flutter. Unnecessary therapies and aggressive interventions may have devastating consequences in these cases. We present a case of a patient with global pseudo-atrial flutter waves in the setting of essential tremor strictly confined to the arms. Two-dimensional transthoracic echocardiography (2D TTE) was used to discriminate normal sinus rhythm from pseudo-atrial flutter waves.

## Introduction

Electrocardiographic artifacts are commonly detected in everyday clinical practice. High-frequency muscle tremor is one of the most common causes of electrocardiographic artifacts, mimicking a variety of supraventricular and ventricular arrhythmias [1-2]. In such cases, a misleading diagnosis and the consequent unnecessary therapies and interventions may have devastating results. Hence, a careful echocardiogram (ECG) examination, followed by other non-invasive imaging techniques if necessary, is of major importance in order to confirm the electrocardiographic diagnosis and decide further therapeutic management [3].

In this case report, we describe the interesting case of a patient with global pseudo-atrial flutter waves in the setting of essential tremor.

## Case presentation

**Figure 1:**
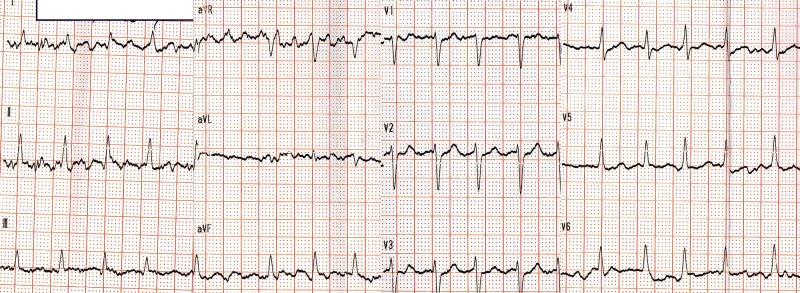
Patient's ECG when admitted to the emergency department ECG: echocardiogram

A 63-year-old female was admitted to the emergency department with a complaint of palpitation, which had started a few hours ago. Her 12-lead ECG was suggestive of atrial fibrillation (AF; Figure 1).

**Figure 2:**
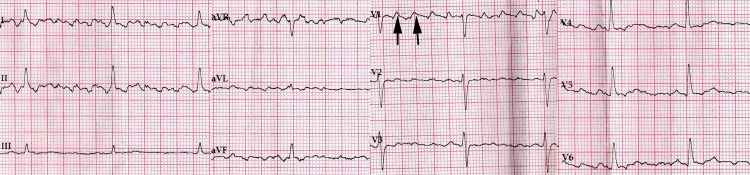
ECG after intravenous administration of amiodarone Patient's arms were immobilized in order to mitigate the presence of potential artifacts due to the tremor. Black arrows show pseudo atrial flutter waves.

The patient’s medical history included oral anticoagulation therapy for recurrent episodes of AF and topiramate due to essential tremor strictly confined to the arms. After intravenous administration of amiodarone, the new 12-lead ECG was compatible with atrial flutter with cycle length 240 ms and 4:1 atrioventricular response (Figure 2).

**Figure 3:**
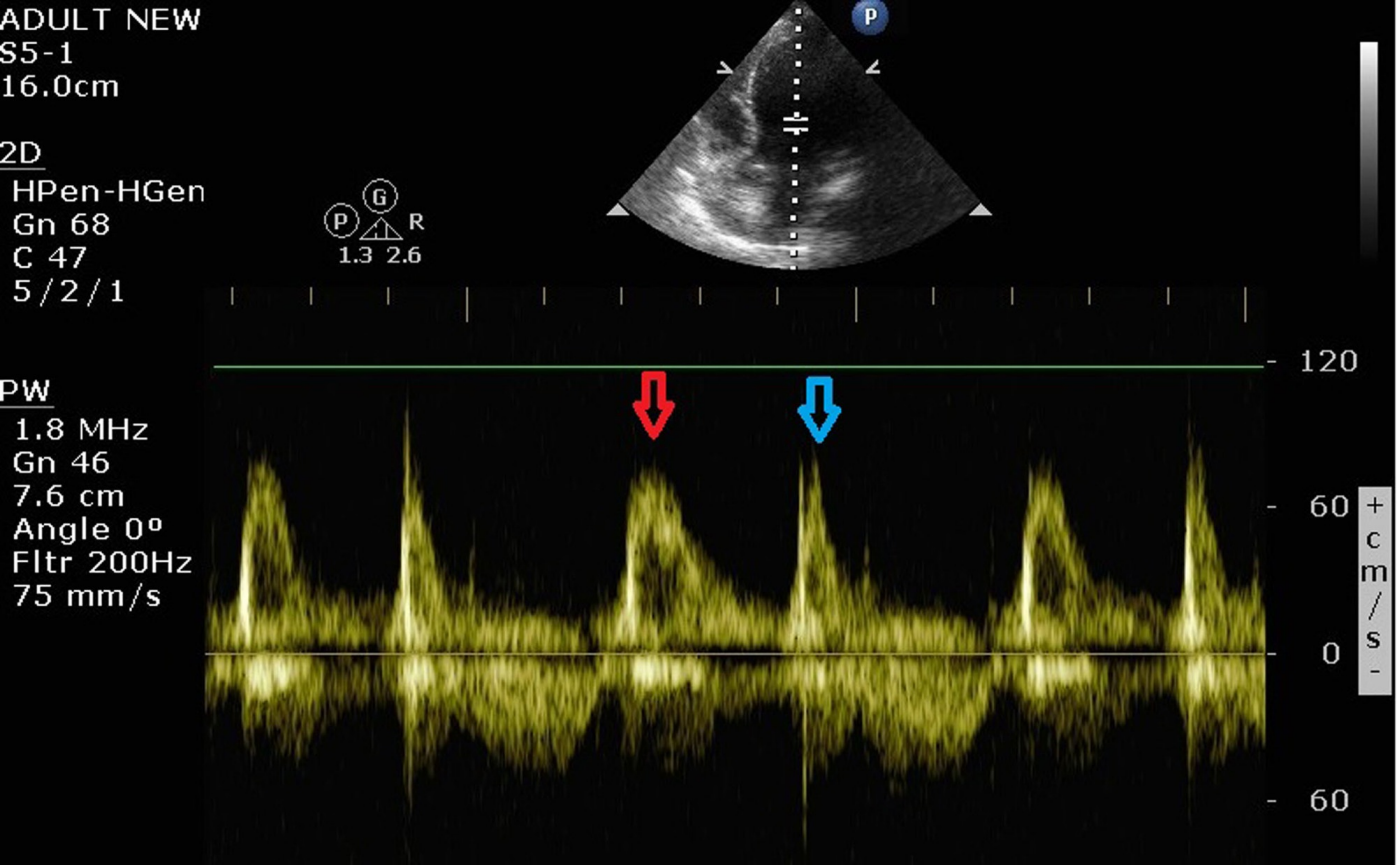
Pulsed wave Doppler revealing a diastolic pattern of normal sinus rhythm Red arrow shows early diastolic E wave and blue arrow late diastolic A wave.

Notably, flutter waves were present in both limb and precordial leads. To evaluate the underlying heart rhythm, two-dimensional transthoracic echocardiography (2D TTE) was performed. Measurement of transmitral flow using pulsed-wave Doppler revealed a diastolic pattern with normal atrial rhythm (Figure 3).

## Discussion

Muscle tremor, hiccups, and medical electrostimulation devices are some of the most common causes of ECG artifacts that can mimic supraventricular tachycardias, including atrial flutter [1,4]. However, in most cases, pseudo-flutter waves are primarily detected in limb leads. We present a case of essential tremor localized in the arms with pseudo-flutter waves in both limb and precordial leads. In recently published case reports, patients with similar clinical presentation were admitted for an electrophysiology (EP) study or multiple ECG records were obtained in order the underlying heart rhythm to be clarified [5]. However, in our patient, multiple ECG records did not differ, even after immobilizing her arms in order to mitigate the potential impact of artifacts. Therefore, we propose the use of 2D TTE for discriminating normal sinus rhythm from pseudo-atrial flutter in such cases, in order imprecise conclusions and unnecessary therapy to be avoided. A transmitral Doppler pattern with an E and A wave or a simultaneous left ventricular inflow and outflow Doppler wave showing a 1:1 atrioventricular relationship are indicative of sinus rhythm. On the other hand, multiple F waves (characteristic M-mode deflections of the mitral leaflets) following an E wave are suggestive of true atrial flutter.

## Conclusions

2D TTE can be easily used to discriminate normal sinus rhythm from pseudo-atrial flutter waves, when ECG examination is inconclusive.
